# Methylation of estrogen receptor 2 (*ESR2*) in deep paravertebral muscles and its association with idiopathic scoliosis

**DOI:** 10.1038/s41598-020-78454-4

**Published:** 2020-12-18

**Authors:** Małgorzata Chmielewska, Piotr Janusz, Mirosław Andrusiewicz, Tomasz Kotwicki, Małgorzata Kotwicka

**Affiliations:** 1 Chair and Department of Cell Biology, Poznan University of Medical Sciences, Rokietnicka Street 5D, Poznan, Poland; 2 Department of Spine Disorders and Pediatric Orthopedics, Poznan University of Medical Sciences, 28 Czerwca 1956 r. Street 135/147, Poznan, Poland

**Keywords:** DNA methylation, Gene expression, Musculoskeletal abnormalities

## Abstract

Idiopathic scoliosis (IS) is one of the most common spinal disorders in adolescents. Despite many studies, the etiopathogenesis of IS is still poorly understood. In recent years, the role of epigenetic factors in the etiopathogenesis of IS has been increasingly investigated. It has also been postulated that the development and progression of the disease is related to gender and puberty, and could be associated with estrogen action. Estrogen hormones act via estrogen receptor 1 (ESR1) and estrogen receptor 2 (ESR2). It has been suggested that *ESR2* expression is dependent on methylation within its gene promoter. So far, no studies have evaluated local, tissue-specific DNA methylation in patients with IS. Thus, our study aimed to analyze the methylation and expression level of *ESR2* in the paraspinal muscles of the convex and concave side of the IS curvature. The methylation level within *ESR2* promoter 0N, but not exon 0N, was significantly higher on the concave side of the curvature compared to the convex side. There was no significant correlation between *ESR2* expression and methylation level in the promoter 0N on the convexity of thoracic scoliosis, whereas, on the concave side of the curvature, we observed a moderate negative correlation. There was no difference in the methylation levels of the *ESR2* promoter and exon 0N between groups of patients with Cobb angle ≤ 70° and > 70° on the concave and convex side of the curvature. We also found no statistically significant correlation between the Cobb angle value and the mean methylation level in either the *ESR2* promoter or exon 0N on the convex or concave side of the curvature. Our findings demonstrate that DNA methylation at the *ESR2* promoter in deep paravertebral muscle tissue is associated with the occurrence but not with the severity of idiopathic scoliosis.

## Introduction

Idiopathic scoliosis (IS) is one of the most common spinal disorders in adolescents, with a reported prevalence of 1–3%. IS is a three-dimensional deformity of the spinal column, resulting in curvature in the coronal plane, sagittal plane deviation, axial rotation of the vertebrae, rib prominence, and trunk imbalance. IS may remain stable throughout an individual's life or could progress to a very severe form. The risk of severe curve progression is associated with the growth of the spine^1^. However, at an early stage of the disease, it is difficult to predict the final phenotype. Severe idiopathic scoliosis leads to cardiorespiratory impairment due to lung restriction and results in significant morbidity. The degree of impairment has been shown to correlate with the severity of the spinal deformity^2–4^. Therefore, identifying patients who are at risk of developing scoliosis and severe curve progression is crucial in order to provide them with early treatment^1,5^.

The idea that IS has a strong genetic background is supported by the higher prevalence of scoliosis in families with an affected member compared to the general population^6^. Candidate genes potentially associated with the occurrence and progression of IS have been identified in many studies, including evaluations of family linkage, genome-wide association studies (GWAS), SNPs (single nucleotide polymorphisms) and gene expression profiling^6^.

The occurrence and progression of IS is related to gender and puberty. The prevalence is much higher, and the curvatures are larger in female adolescents compared to males. Thus, it has been postulated that IS progression could be influenced by estrogen action^7–9^. Estrogen hormones act on target cells through two types of receptors: estrogen receptor 1 (ESR1) and estrogen receptor 2 (ESR2)^10,11^ and through G protein–coupled estrogen receptor 1 (GPER), which is a membrane protein^12^. It was postulated that two SNPs of the *ESR1* were associated with IS^13–17^. However, the relationship between *ESR1* polymorphisms and IS remains ambiguous^18–20^. Meta-analysis of four studies performed by Chen et al*.* revealed a non-significant association between rs9340799 *ESR1* polymorphism and AIS^21^. The initial results regarding the association of the *ESR2* polymorphism rs1256120 with a susceptibility to occurrence and progression of IS were not confirmed in replication studies^19,22,23^. Nevertheless, there is a suggestion that other *ESR2* polymorphisms may be associated with IS curvature progression to the severe forms^24^. Additionally, the association of *GPER* polymorphisms with severity of the curve in patients with idiopathic scoliosis was demonstrated. Abnormalities in GPER presence may influence the deterioration of spine deformity^12^. However, it was not confirmed in replication study^25^.

In recent years, an increasing number of reports have described the effect of estrogens on skeletal muscle^26,27^. ESR2 has been detected at both mRNA and protein levels in skeletal muscle^28^. *ESR2* expression was also observed in paravertebral skeletal muscle tissue^26^.

However, the polymorphisms associated with IS are commonly present in the DNA of individuals without IS as well^6^. Therefore, it has been postulated that IS arises from both genetic and environmental factors. Based on observations from twin studies, Grauers et al. estimated that the risk of developing scoliosis is the result of an additive genetic effect in the minority, and the result of environmental effects in the majority of affected individuals^29^. Cheng et al. suggested that genetics play a more significant role during the initiation phase of adolescent idiopathic scoliosis (AIS), whereas during curve progression, environmental factors have a greater contribution^30^. The identification of the factors linking the genome and the environment in IS etiopathogenesis is crucial in the prevention and treatment of the disease. One mechanism often cited as a link between genetics and the environment is epigenetics. However, investigating the potential epigenetic mechanisms that underlie IS etiology is a relatively new avenue of research^31,32^. To date, only a few studies describing DNA methylation in IS were published^32–36^. Additionally, none of the studies published so far evaluated local, tissue-specific DNA methylation.

DNA methylation is an epigenetic DNA modification associated with the regulatory regions of some genes. It has been suggested that *ESR2* expression in disease contexts is influenced by methylation at 0N and 0K gene promoters and their corresponding exons^37^. Differences in methylation levels between patients with different IS phenotypes could be associated with disease susceptibility or progression. Thus, we sought to analyze the methylation and expression levels of *ESR2* in the paraspinal muscles of the convex and concave side of the IS curvature.

## Results

### Patient characteristics

The age of patients at the surgery ranged from 12.1 to 17.9 years, mean 14.5 ± 1.5 years. The mean Cobb angle was 77.4 ± 16.1°, with a range from 52° to 115°. There was no difference between patients with Cobb angle ≤ 70° *versus* patients with Cobb angle > 70° in mean age (14.5 ± 1.3 *vs*. 14.7 ± 1.7, *P* = 0.9), number of curvatures (3 single: 7 double *vs.* 8 single: 11 double, *P* = 0.7) and Risser sign (median 4 *vs.* 4, *P* = 0.7). The main difference between the groups was in the Cobb angle value 61.1° ± 6.0° *vs.* 86° ± 12.7° (*P* < 0.0001).

### DNA methylation at the *ESR2* promoter 0N

**Figure 1:**
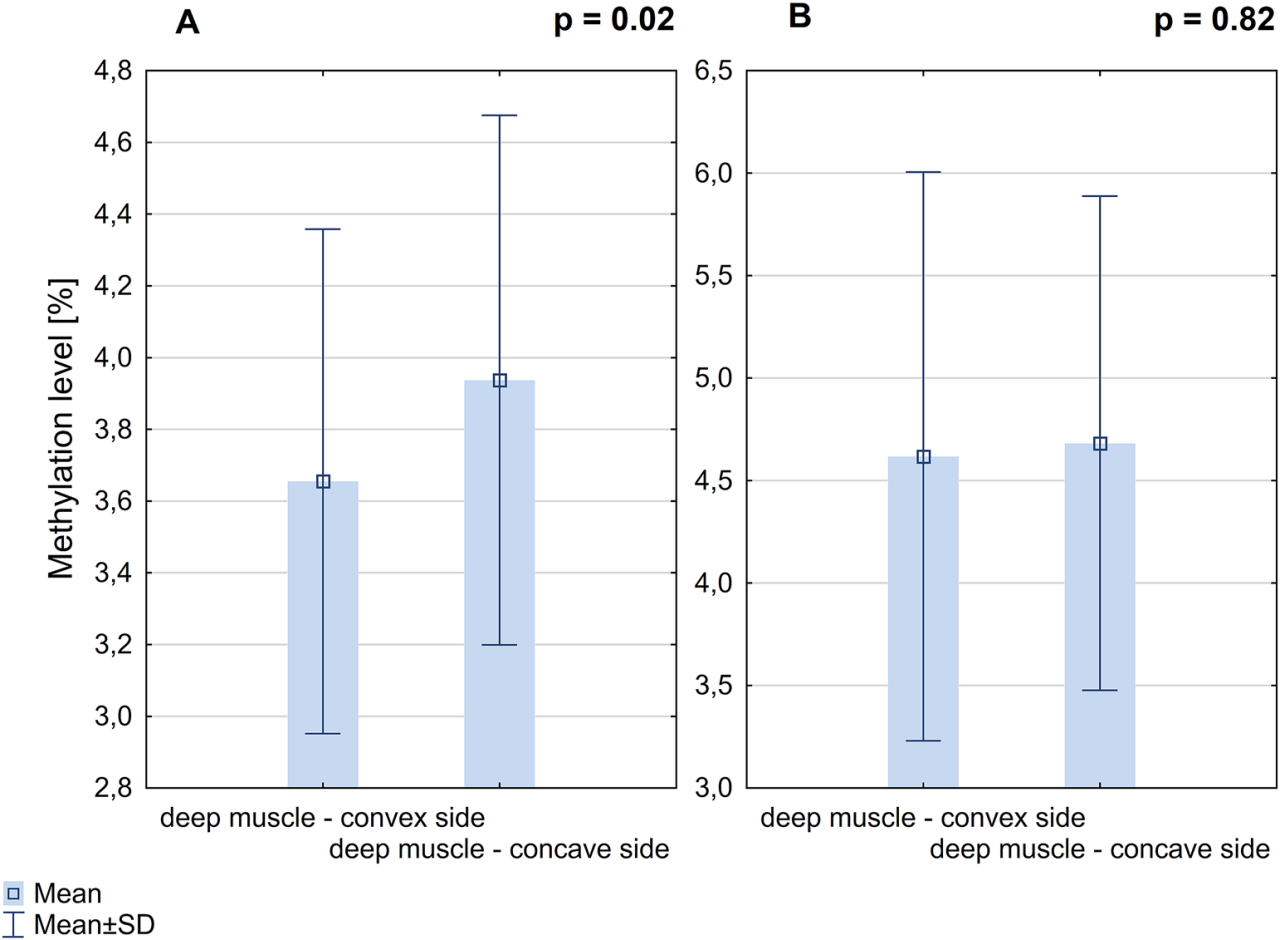
DNA methylation level within *ESR2* promoter 0N (**A**) and exon 0N (**B**) in deep paravertebral muscles.

**Figure 2:**
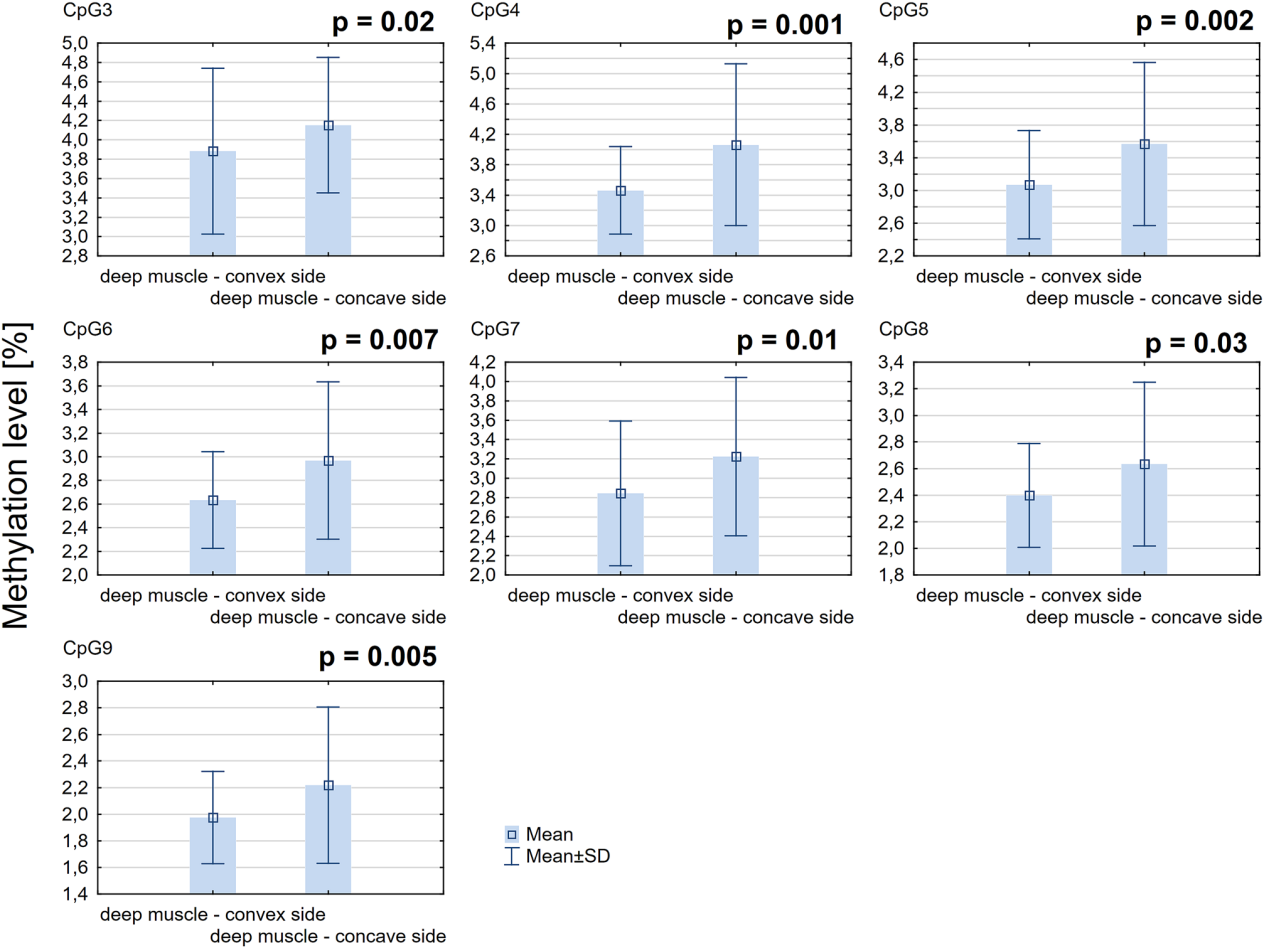
DNA methylation level in seven CpG sites localized within *ESR2* promoter 0N in deep paravertebral muscles.

The mean methylation level of all CpG sites within *ESR2* promoter 0N was significantly higher on the concave side of the curvature comparing to the convex side (3.94% ± 0.74% *vs.* 3.66% ± 0.7%; *P* = 0.02; Fig. 1A). The methylation level of seven CpG sites within *ESR2* promoter 0N (each CpG analyzed separately) was significantly higher on the concave side of the curvature compared to the convex side (*P* < 0.05, Additional file 1: Table S1; Fig. 2).

### DNA methylation level of the *ESR2* exon 0N

The mean methylation level of all CpG sites within *ESR2* exon 0N was not significantly different between deep paravertebral muscle on the convex side of the curvature *vs.* concave side (4.62 ± 1.39 *vs.* 4.68 ± 1.21; *P* = 0.82; Fig. 1B). Additionally, there was no significant difference in the methylation level at each CpG site (each CpG analyzed separately) between deep paravertebral muscles on the convex and concave side of the curvature (*P* > 0.05, Additional file 1: Table S2).

### Correlation between *ESR2* methylation levels and relative expression of the *ESR2* gene

**Figure 3:**
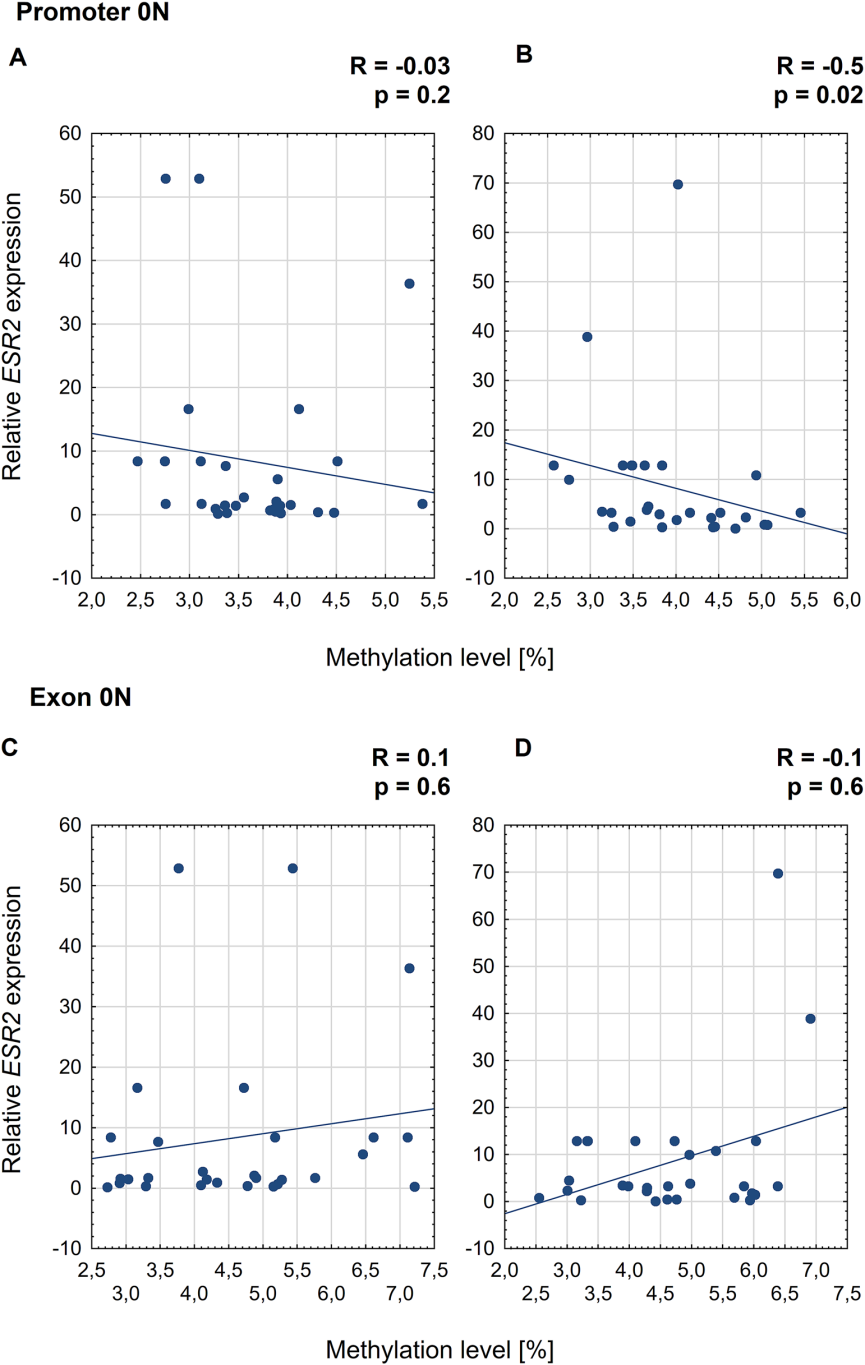
Correlation between *ESR2* expression and mean methylation level in promoter 0N and exon 0N on the convex (**A**,**C**) and concave (**B**,**D**) side of the curvature.

**Figure 4:**
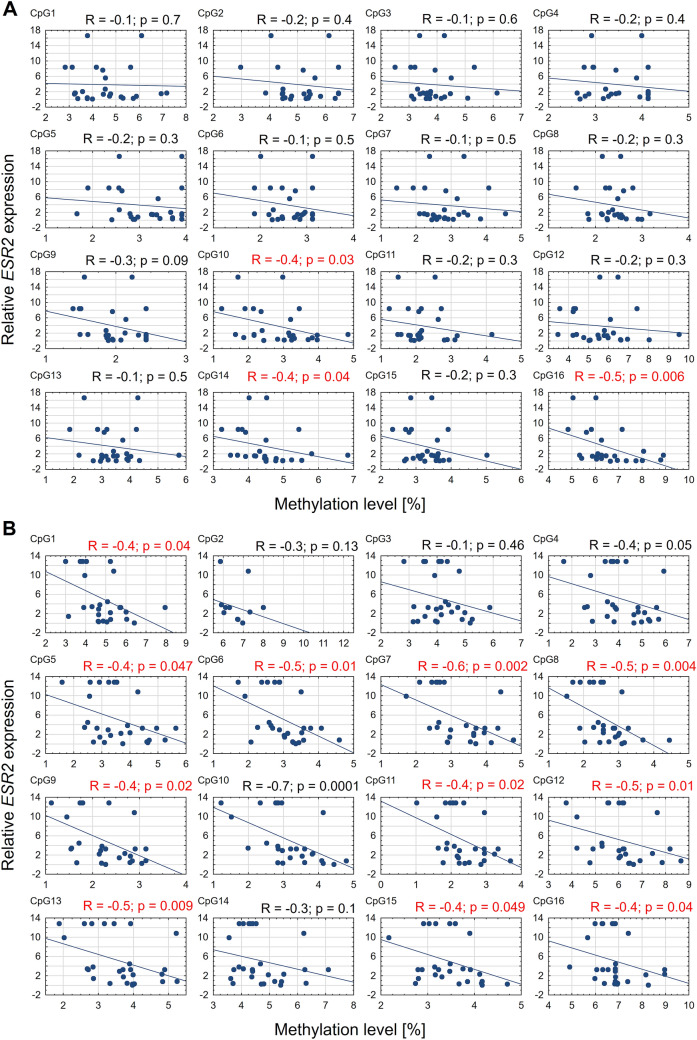
Correlation between *ESR2* expression and methylation level at each CpG site in promoter 0N on the convex (**A**) and concave (**B**) side of the curvature.

**Figure 5:**
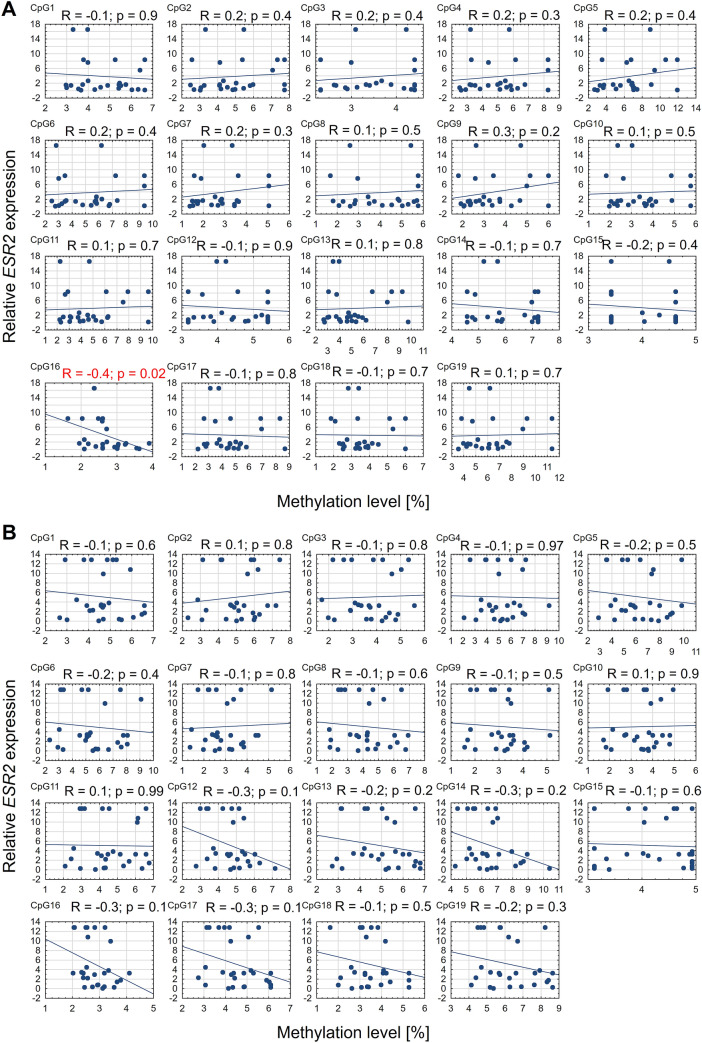
Correlation between *ESR2* expression and methylation level at each CpG site in exon 0N on the convex (**A**) and concave (**B**) side of the curvature.

On the convex side of thoracic scoliosis, the correlation between *ESR2* expression and mean methylation level in promoter 0N was weak, negative, and non-significant (Spearman's rank correlation, R = − 0.3; *P* = 0.2; Fig. 3A). In contrast, on the concave side of the curvature, a moderate negative correlation was observed (R = -0.5; *P* = 0.02; Fig. 3B). Additionally, a significant negative correlation between *ESR2* mRNA expression and methylation level was observed at 3 and 11 CpG sites in promoter 0N on the convex and concave side of the curvature, respectively (R ranged from − 0.4 to − 0.6, *P* < 0.5; Fig. 4). In the case of the exon 0N region, the significant, negative correlation between *ESR2* expression and methylation level was observed at only one CpG site on the convexity of thoracic scoliosis (R = − 0.4; *P* = 0.02; Fig. 5). The correlation between *ESR2* expression and mean methylation level in exon 0N was weak, positive, non-significant on the convex side of the curvature (Spearman's rank correlation: R = 0.1; *P* = 0.6; Fig. 3C) and weak, negative, non-significant on the concavity of thoracic scoliosis (Spearman's rank correlation: R = -0.1; *P* = 0.6; Fig. 3D).

### Association between methylation status of *ESR2* and Cobb angle

**Figure 6:**
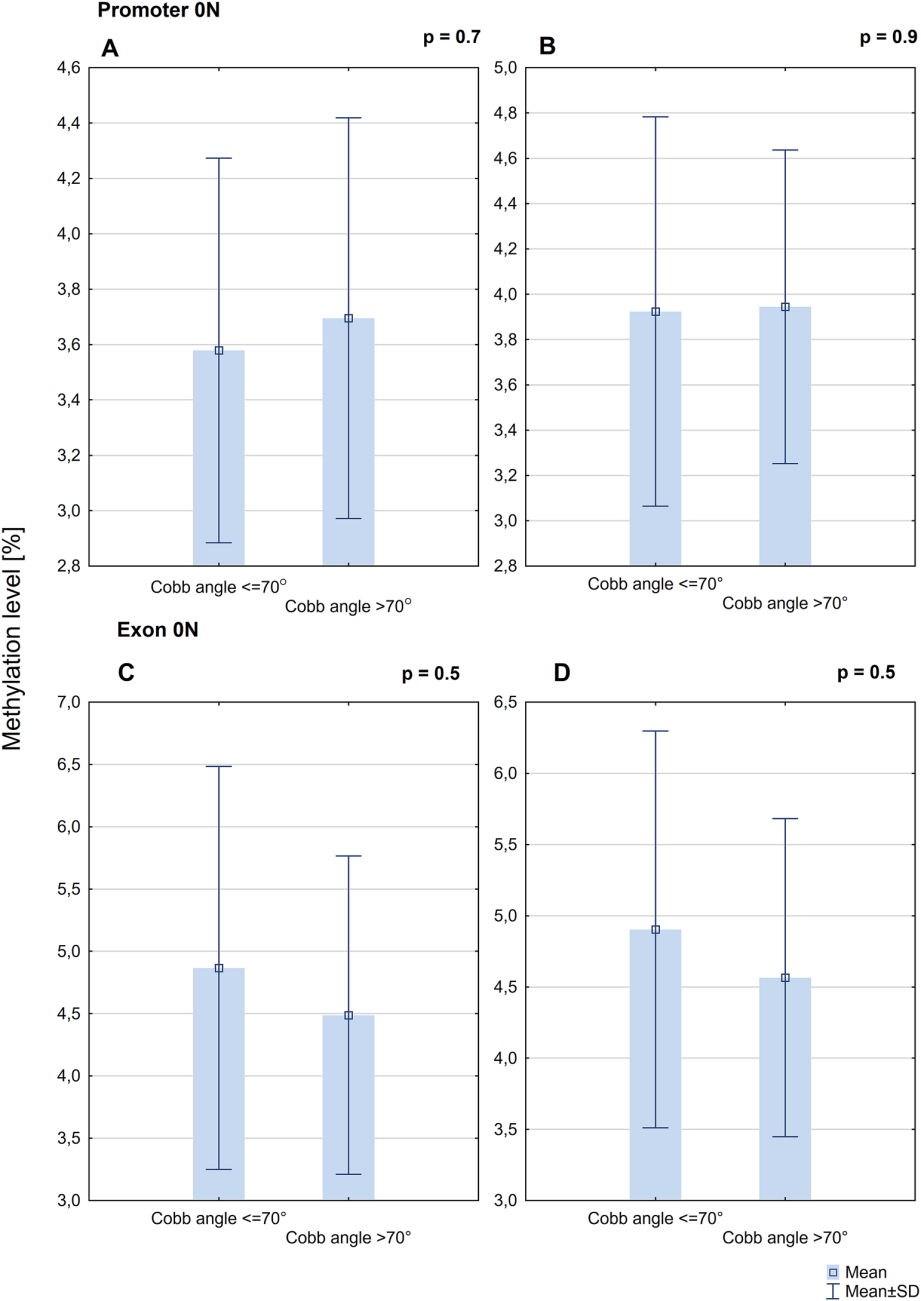
DNA methylation level within *ESR2* promoter 0N on the convex (**A**) and concave side of the curvature (**B**) and exon 0N on the convex (**C**) and concave (**D**) side of the curvature in the group of patients with Cobb angle ≤ 70° and > 70°.

There was no difference in *ESR2* promoter 0N mean methylation level between groups of patients with Cobb angle ≤ 70° (n = 10) and > 70° (n = 19) on the concave (3.58% ± 0.69% *vs.* 3.70% ± 0.72%; *P* = 0.7; Fig. 6A) and convex (3.92% ± 0.86% *vs.* 3.94% ± 0.69%; *P* = 0.9; Fig. 6B) side of the curvature. There was no difference in *ESR2* exon 0N mean methylation levels between the group of patients with Cobb angle ≤ 70° and the group of patients with Cobb angle > 70° on the concave (4.86% ± 1.62% *vs* 4.49% ± 1.28%; *P* = 0.5; Fig. 6C) and convex (4.90% ± 1.39 *vs* 4.57% ± 1.12%; *P* = 0.5; Fig. 6D) side of the curvature.

There was no statistically significant correlation between Cobb angle value and mean methylation level in the *ESR2* promoter 0N on the convex (Pearson’s correlation test, R = 0.3, *P* = 0.2) and concave side (R = 0.1, *P* = 0.8) of the curvature. The results for exon 0N were similar to those for promoter 0N (convexity of thoracic scoliosis: R = − 0.1, *P* = 0.7; concavity of thoracic scoliosis: R = − 0.1, *P* = 0.5).

## Discussion

The etiopathogenesis of idiopathic scoliosis remains poorly understood^6^. One of the biggest challenges of research into IS etiopathogenesis is that affected individuals possess no obvious structural deficiencies or malformations in the vertebral column and associated soft tissues, except the curvature of the spine^38^.

Current views considered IS as a multifactorial disease with predisposing genetic factors^39^. Many factors contributing to the occurrence of IS were described, such as mechanical, hormonal, metabolic, neuromuscular, growth, and genetic abnormalities. Amongst these, some factors may be epiphenomena rather than the cause itself. Other factors may contribute to curve progression, rather than curve initiation^40^.

By studying two sides of the paravertebral muscles of IS patients, we aimed to identify the molecular background for AIS-associated paravertebral muscle changes.

The development and growth of the skeleton are controlled by parathyroid, thyroid hormones, and growth hormones, but also by estrogens^41^. It has been reported that factors associated with puberty and sexual dimorphism play an important role in the pathogenesis of idiopathic scoliosis. However, results demonstrating this relationship are ambiguous. Kulis et al. observed that blood estrogen levels in female patients with idiopathic scoliosis are significantly lower compared to unaffected individuals^42^. Conversely, Raczkowski and colleagues found that the level of circulating estrogens in the blood did not differ significantly between females in a control group and the group of patients with IS^43^. Although the role of estrogens in IS has not been fully uncovered, it has been suggested that estrogen-mediated signal transduction may be dysregulated in affected cells^27,43^.

In previous studies, we observed asymmetric expression of *ESR2* in deep paravertebral muscles on the concave and convex side of the curvature. The relative expression of the *ESR2* gene correlated with Cobb angle values and the risk factor of progression^26^. However, the mechanism of estrogen action in IS has yet to be established. In recent years, the role of epigenetic factors in the etiopathogenesis of IS has been increasingly investigated^32,44^. *ESR2* expression may be regulated by methylation of CpG islands within the 0N and 0K promoters and their corresponding exons, which generate transcript variants that differ in their 5′UTR (5′ untranslated) regions^37,45^. However, there has yet to be an assessment of DNA methylation at *ESR2* regulatory regions in the deep paravertebral muscles of patients with idiopathic scoliosis.

We observed that methylation levels within *ESR2* promoter 0N, but not exon 0N, were significantly higher on the concave side of the curvature compared to the convex side. The difference in methylation levels in deep paravertebral muscles may be associated with their heterogeneous histological architecture^46^. Muscle tissue on the concave side was characterized by a higher content of fibrous elements. Moreover, the ratio of slow-twitch to fast-twitch fibers is shifted, with a higher prevalence of fast-twitch fibers on the concave side of the curvature^47^. What is more, higher electromyographic activity in paravertebral muscles on the convex side than on the concave side was reported. This phenomenon was studied in relation with potential treatment possibilities and predictive value^48,49^. According to Weiss, this asymmetry can be reduced with specific exercises^50^.

However, it is difficult to distinguish the causative factors from their effects. The difference in methylation may be the cause of the asymmetry in muscles, which may have some contribution to the etiology of IS. Conversely, differences in methylation levels could be a consequence of the muscles being exposed to different conditions on either side of the curvature due to asymmetric loading or other unknown conditions. Taking into consideration that the curvature size did not correlate with the methylation level, the asymmetrical loading is likely not the reason for the difference in methylation level, because the asymmetry of loading transmission increases with the curvature size.

Postural control deficits in patients with IS was described by Sahlstrand et al*.* and confirmed in more recent publications and seems to be a repeatable observation^51,52^. When discussing the postural imbalance in IS, neurophysiological function and structure of muscles, the theory of a functional tethering of the spinal cord as a possible background factor for IS needs to be mentioned. Chu et al. described the relative shortening and functional tethering of spinal cord in IS patients. They found an increased prevalence of abnormal somatosensory evoked potentials and the low-lying cerebellar tonsil in severe IS and compared with mild to moderate IS. Similar observations concerning the association of IS with the severe curve with tonsillar ectopia and abnormal somatosensory function were described by Cheng et al*.*^53^. Deng et al. postulated the cord-vertebral length ratio as a significant predictor for curve progression in IS^54^. Taking into consideration the impact of the functional spinal cord tethering on the signal transduction this phenomenon may affect both function and histological structure of the paraspinal muscles.

It is widely accepted that epigenetic modifications can impact gene expression^55^. Consistent with this, we found an association between the expression of *ESR2* and promoter 0N methylation. Specifically, *ESR2* expression negatively correlated with methylation level, which is consistent with the phenomenon of methylation-dependent gene silencing. These results provide further support for the hypothesis that 0N promoter methylation modulates *ESR2* gene expression levels in the deep paravertebral muscle tissues in the case of patients with IS.

The phenotypic heterogeneity of IS makes it challenging to form an efficient strategy to prevent the disease or its progression^56^. The phenotype of IS differs vastly between patients. Our division of IS phenotypes according to curve severity was based on clinical studies^57–59^. According to clinical practice, IS between 50º and 70º requires surgeon intervention due to the risk of further progression. The aim of surgery in such cases is to avoid future complications due to possible progression^57–59^. On the other hand, in more severe scoliosis, significant health problems associated with lung function, cardiac function, and back pains can occur^2,4,58^.

Understanding the etiopathogenetic background of IS susceptibility as well as factors enhancing curvature progression has crucial clinical implications. Identification of the patients who are most at risk of curve progression allows clinicians to modify the treatment approach. Currently, no defined threshold marks when severe scoliosis produces a significant impact on a patient's health. Most studies concerning surgical treatment of scoliosis classify severe curvature as a Cobb angle exceeding 70°^60–62^. Thus, we applied this value to categorize study groups.

To our knowledge, this study is the first to evaluate DNA methylation in muscle tissues affected by IS. Previous studies did not consider locally acting factors, instead focusing on global markers^32–36^. We found just one study investigating epigenetic changes in muscle tissue in IS patients. Jiang et al. evaluated the involvement of non-coding RNA in IS-affected tissue^63^. It is difficult to compare our results directly since we investigated different epigenetic mechanisms. Nevertheless, both studies support the importance of evaluating local changes in the affected region of the body to find causative agents of disease.

Several attempts have been made to evaluate the importance of DNA methylation in IS etiopathogenesis. Mao et al. analyzed the association of five CpG sites of cartilage oligomeric matrix protein gene (*COMP*) and its expression with IS^34^. Shi et al*.* published two studies concerning DNA methylation in AIS^35,36^. Both the studies by Shi et al. and Mao et al. were performed on peripheral blood samples of AIS patients and compared to healthy controls. Additionally, Meng et al. performed methylation analysis of the whole genome in two pairs of monozygotic twins and observed that more severe curvature was associated with decreased methylation at site cg01374129 on chromosome 8^32^. Liu et al. also carried out a whole-genome methylation analysis in twins. They found a significantly higher methylated region in chromosome 15 in the AIS group compared to the controls^33^. All these studies provide essential information about the role of DNA methylation in IS etiopathogenesis. However, the chosen tissue (peripheral blood) limited these findings to the evaluation of globally acting factors.

A strength of our study is that we performed a comprehensive evaluation of pathology at the muscular origin of the disease. Thus, our data add valuable insights into the DNA methylation and its biological action in the background of IS. Another key feature of the study is the analysis of IS phenotypes. Although we did not identify an association between methylation and IS severity, our results support the theory that factors associated with occurrence and progression of IS differ considerably.

Finally, we acknowledge that our study also had some limitations. Firstly, our investigation was limited by the sample size. However, the number of patients is comparable to other published research investigating DNA methylation in IS^32–36^. The study was also limited by the lack of tissue samples obtained from healthy controls. We decided not to acquire control samples from individuals who underwent surgery due to degenerative spine disease. This decision was taken because such patients are mostly elderly, and chronic degenerative disease of the spine may cause the muscles atrophy, or induce unknown methylation changes. Similarly, it would be beneficial to compare our results in the case of patients with curves between 20 and 30° to evaluate the impact of *ESR2* methylation on IS progression. However, such patients do not need surgical treatment, and there is no possibility to obtain muscle samples.

## Conclusions

In conclusion, our results demonstrate that the methylation pattern of CpG sites in the regulatory regions of the *ESR2* gene in the deep paravertebral muscle tissue is associated with the occurrence but not with the severity of idiopathic scoliosis. The tissue samples obtained from patients who underwent surgery due to IS showed substantial differences in methylation levels at *ESR2*. Altogether, the presented findings suggest that differences in methylation levels at the concave compared to the convex side of the spinal curvature may be associated with IS etiopathogenesis. The relationship between the promoter and exon 0N methylation levels and *ESR2* gene expression indicates that this type of epigenetic modification may affect the tissue-dependent response to estrogens.

## Methods

The study was approved by the Institutional Review Board of the Poznan University of Medical Sciences (No 546/17 and 741/19). Informed consent was obtained from all the patients or their parents/legal guardians in the case of under-aged participants. All methods were carried out in accordance with the approved guidelines.

### Patients

Twenty-nine girls with severe IS were included in the study. All patients underwent posterior spinal surgery in one hospital in a Central European country (Poland) from January 2017 until December 2019. Patients were subject to a clinical, radiological, and molecular examination. All patients had undergone standing posteroanterior X-rays before surgery. The curve pattern (number and localization of the curvatures), Cobb angle (angle of curvature size)^64^, and Risser sign (the radiological sign of skeletal maturity)^65^ were measured by an experienced spine surgeon.

The inclusion criteria were as follows: (1) clinically and radiologically confirmed IS diagnosis, (2) no coexisting orthopedic, genetic or neurological disorders, (3) primary thoracic spinal curvature (4) surgical treatment due to IS. The patients were divided into groups according to disease severity. The first group consisted of ten patients with a moderate form of idiopathic scoliosis with curvature ranging from 50° to 70° and a Risser sign of ≥ 3 or age ≥ 15 years old. The second group consisted of nineteen patients with a very progressive form of idiopathic scoliosis with larger curvatures exceeding 70°, regardless of Risser sign or age.

### Tissue samples

During the surgery, two muscle tissue fragments were obtained from each patient. These were from the deep paravertebral muscles (*m. longissimus*) on the (1) convex and (2) concave side of the curvature (1 cm^3^ each). Samples were stored in sterile tubes containing 5 ml nucleic acid stabilizing solution (Novazym).

### Genomic DNA methylation analysis

#### Genomic DNA isolation

Genomic DNA was extracted using Quick-DNA Miniprep Plus Kit (Zymo Research) according to the manufacturer's protocol with modifications. Firstly, tissue samples were ground in liquid nitrogen with a mortar and pestle. Then, 25 mg were incubated overnight at 55 °C with proteinase K. Next, the lysate was centrifuged to remove insoluble debris. At this point, isolation was continued following the steps outlined in the protocol. The DNA quantity and purity were assessed using a spectrophotometer (NanoPhotometer NP80, Implen) by measuring absorbance at A = 260, A = 230 nm, and ratios A = 260/230, A = 260/280. All analyzed samples met the criteria for purity (both ratios ranged from 1.9 to 2.0). DNA integrity was evaluated using a standard 1% agarose gel (Lab Empire) electrophoretic separation in the presence of ethidium bromide (50 ng/ml, Merck).

#### Bisulfite conversion

One microgram of genomic DNA was bisulfite converted using EZ DNA Methylation Kit (Zymo Research) following the manufacturer's protocol and eluted with 10 µl of M-Elution Buffer.

#### Polymerase chain reaction and pyrosequencing analysis

Pyrosequencing reactions were preceded by polymerase chain reaction (PCR) with bisulfite converted DNA as the template. Specific primers for both reactions were designed using PyroMark Assay Design 2.0 software (Qiagen) and synthesized by Genomed. The input DNA sequences corresponded to the promoter 0N and exon 0N regions of *ESR2* gene reference sequences deposited in the Nucleotide Database of National Center for Biotechnology Information (https://www.ncbi.nlm.nih.gov; GenBank No.: NG_011535.1). Sequencing, forward, and reverse primers are presented in Table 1. PCR was performed in a total volume of 10 µl using ZymoTaq PreMix (Zymo Research) designed for the amplification of bisulfite-treated DNA (Table 2). 2 µl of the products were analyzed using standard 2% agarose gel (Lab Empire) electrophoretic separation, and compared to Nova 100 mass marker (Novazym) in the presence of ethidium bromide (50 ng/ml, Merck).

**Table 1:** Primer sequences and location.

	Primer	Sequence		Tm (°C)	GC (%)	PCR product size	Location with respect to TSS	Location with respect to ATG
*ESR2* promoter 0N	→PCR	GGTATTTTTTAGGATTTGGTTGGAAATGTA	30	60,9	30,0	276 bp	− 239	− 11,663
	←PCR^B^	ACTTAACCATAAACCCCTTCTTCCTTT	27	58,9	37,0		+ 37	− 11,387
	SEQ	ATATTTTTAGGTTTTATTTTAGAT	24	40,7	12,5	–	− 209	− 11,633
*ESR2 *exon 0N	→PCR	GGAGGTTGAGAGAAATAATTGTTTTTTGA	29	57,7	31,0	253 bp	+ 115	− 11,309
	←PCR^B^	AAACACACCCACCTTACCTTCTCTA	25	58,3	44,0		+ 368	− 11,057
	SEQ	GTTTTTTGAAATTTGTAGGG	20	44,8	30,0	–	+ 135	− 11,289

→PCR, forward primer;  ←PCR, reverse primer; ^B^, biotinylated primer; Tm, melting temperature, GC, guanine-cytosine content; bp, base pairs; TSS, transcription start site; ATG, start codon; SEQ, sequencing primer.

**Table 2:** PCR mixture content and thermal profile of the reactions.

PCR reaction mixture				
Component	Initial concentration	Volume added	Final concentration	Mixture volume
ZymoTaq™ Premix	2x	5 µl	1x	10 µl
→PCR	10 µM	1 µl	1 µM	
←PCR	10 µM	1 µl	1 µM	
DNA	100 ng/µl	0,2 µl	2 ng/µl	
nuclease-free water		2,8 µl		
**Thermal profile of the reactions**				
**Number of cycles**	**Step**		**Duration, temperature**	
1	initial denaturation		10 min., 95 °C	
37	denaturation		30 s., 95 °C	
	annealing		30 s., 54 °C	
	extension		60 s., 72 °C	
1	final extension		7 min., 72 °C	
1	hold		∞, 4 °C	

→PCR, forward primer; ←PCR, reverse primer; min., minutes, s., seconds.

Pyrosequencing analysis was performed using the PyroMark Q48 instrument (Qiagen). CpG assays were designed using Pyromark Q48 Autoprep 2.4.2 software (Qiagen). We analyzed 16 and 19 CpG sites for promoter and exon 0N, respectively. Each reaction contained an internal control that allowed to assess whether the sodium bisulfite treatment was successful. Methylation levels were quantified using Pyromark Q48 Autoprep 2.4.2 software (Qiagen) and determined as a percentage ratio of methylated to non-methylated dinucleotides.

### Analysis of *ESR2* mRNA expression level

#### Total RNA isolation

Total cellular RNA was extracted using RNA Isolation Reagent (GenoPlast Biochemicals) and Direct-zol RNA Miniprep Kit (Zymo Research) according to the manufacturer's protocol with modifications. Firstly, 25 mg of powdered muscle tissue was transferred to RNA Isolation Reagent and incubated at room temperature for 5 min (min). Next, 200 µl of chloroform was added, and samples were shaken vigorously and incubated at room temperature for 3 min. Then, samples were centrifuged (12,000×g for 15 min at 4 °C), and the upper aqueous phase was subsequently transferred to an equal volume of absolute ethanol. At this point, isolation was continued following the steps outlined in the protocol. The RNA sample quantity and purity were assessed using a spectrophotometer (NanoPhotometer NP80, Implen). All analyzed samples met the criteria for purity. RNA integrity was evaluated by 18S and 28S ribosomal RNA bands presence observation, using a standard denaturating 1% agarose gel (Lab Empire) electrophoretic separation in the presence of ethidium bromide (50 ng/ml, Merck).

#### Reverse transcription reaction

cDNA (complementary DNA) synthesis was performed using Expand Reverse Transcriptase (Roche) according to the manufacturer's protocol with modifications described below. The total volume of reaction was 10 µl. In the first step, the mixture containing 500 ng of total RNA, DNase, RNase, pyrogen-free water, 5 mmol/μl universal oligo (dT)10 primer (Genomed) and 300 nmol/µl random hexamer primer (Genomed) was prepared and denatured at 65 °C for 10 min then cooled on ice. Next, 2 mmol/μl of each deoxynucleotide triphosphates (dNTPs, Solis BioDyne), 1.5 U/reaction of E.coli Poly(A) Polymerase (Carolina Biosystems), 150 nm/µl deoxyadenosine triphosphates (dATPs, Carolina Biosystems), 15U /reaction of ribonuclease inhibitor (RNase Inhibitor, Roche), 1 × buffer (Expand Reverse Transcriptase buffer; Roche), and 10 U/reaction of reverse transcriptase (Expand Reverse Transcriptase, Roche) were added. Samples were incubated at 25 °C for 10 min, 55 °C for 60 min, then 5 min at 85 °C. cDNA was either immediately used for quantitative polymerase chain reaction (qPCR) or stored at − 20 °C until further analysis (but no longer than seven days).

#### Quantitative polymerase chain reaction

Quantitative analysis of *ESR2* mRNA expression was evaluated using a hydrolysis probe PrimePCR (qHsaCEP0052206, BioRad). The hypoxanthine–guanine phosphoribosyltransferase (*HPRT*) gene was used as a reference gene (RealTime ready *HPRT*, 102079, Roche). The total volume of 20 µl reaction mixture contained 5 µl of cDNA, 1 × LightCycler FastStart TaqMan Probe Master (Roche), 1 × *ESR2* PrimePCR or 1 × RealTime ready *HPRT,* and nuclease-free water. qPCR reactions were performed using the LightCycler 2.0 carousel glass capillary-based system (Roche). The thermal profile of the qPCR reaction was as follows: pre-incubation step at 95 °C for 10 min, 45 quantification cycles (denaturation at 95 °C for 10 s, annealing/extension step at 60 °C for 30 s, and the final step at 72 °C for 1 s (fluorescence level acquisition mode) and the final cooling to 40 °C for 30 s. Each sample was analyzed in duplicate with independently synthesized cDNA. The quantitative PCR results were assembled using the LightCycler Data Analysis (LCDA) Software version 5.0.0.38, and the obtained fluorescence measurement results were normalized to standard curves. In each sample, *ESR2* expression levels were compared to the reference gene expression level to obtain Cr value (concentration ratio), which corresponded to the relative *ESR2* expression level.

### Statistical analysis

Statistical analysis was performed using Statistica 13.3 software (TIBCO Software Inc.). The methylation level of evaluated CpG sites was analyzed in two ways: together as a mean value of the all chosen CpG sites in promoter 0N or exon 0N, and separately for each CpG site in each region. Data are presented as mean ± SD (standard deviation) and considered statistically significant when *p* < 0.05. The Shapiro–Wilk test was used for the normality of continuous variables distribution assessment. A paired sample t-test or Wilcoxon matched-pairs signed-rank test was applied to compare the methylation levels in deep paravertebral muscles. The correlation coefficients were determined by Pearson's (r) or Spearman's (R) tests. The methylation levels between groups of patients with Cobb angle ≤ 70° and > 70° were compared using an independent t-test.

### Ethics statement

The study was approved by the Institutional Review Board of the Poznan University of Medical Sciences (No 546/17 and 741/19). Informed consent was obtained from all the patients or their parents/legal guardians in the case of under-aged participants.

## Supplementary information

Supplementary Information.

## Data Availability

The datasets used and analyzed during the current study are available from the corresponding author on reasonable request.

## Abbreviations

5′UTR: 5′ Untranslated region
AIS: Adolescent idiopathic scoliosis
cDNA: Complementary DNA
COMP: Cartilage oligomeric matrix protein
dATP: Deoxyadenosine 5′-triphosphate
DNase: Deoxyribonuclease
dNTP: Deoxyribonucleoside triphosphates
ESR1: Estrogen receptor 1
ESR2: Estrogen receptor 2
GPER: G protein-coupled estrogen receptor 1
GWAS: Genome-wide association study
HPRT: Hypoxanthine-guanine phosphoribosyltransferase
IS: Idiopathic scoliosis
LCDA: LightCycler data analysis
PCR: Polymerase chain reaction
qPCR: Quantitative polymerase chain reaction
RNase: Ribonuclease
SD: Standard deviation
SNP: Single nucleotide polymorphism
