# Supplementary material for: Methylation of estrogen receptor 2 (ESR2) in deep paravertebral muscles and its association with idiopathic scoliosis
Source: Sci Rep. 2020 Dec 18;10:22331. doi: 10.1038/s41598-020-78454-4 (PMC7749113; doi:10.1038/s41598-020-78454-4)
Supplement: Supplementary file 1 — Supplementary Information. [file 41598_2020_78454_MOESM1_ESM.docx]

**Methylation of estrogen receptor 2 (*ESR2*) in deep paravertebral muscles and its association with idiopathic scoliosis**

**Małgorzata Chmielewska^1,†*^** (ORCID: 0000-0003-0091-6492), **Piotr Janusz^2,†^** (ORCID: 0000-0001-5702-4933)**, Mirosław Andrusiewicz^1^** (ORCID: 0000-0002-8781-3447), **Tomasz Kotwicki^2^** (ORCID:0000-0003-0810-9361), **Małgorzata Kotwicka^1^** (ORCID: 0000-0002-9802-374X)

^1^ Chair and Department of Cell Biology, Poznan University of Medical Sciences, Rokietnicka Street 5D, Poznan, Poland

^2^ Department of Spine Disorders and Pediatric Orthopedics, Poznan University of Medical Sciences, 28 Czerwca 1956 r. Street 135/147, Poznan, Poland

^†^Contributed equally

^*^Correspondence: mchmielewska@ump.edu.pl

Table S1. Methylation level [%] of 16 CpGs within *ESR2* promoter 0N (N=29).

|  | Min | Max | Mean | SD | *P*-value |
| --- | --- | --- | --- | --- | --- |
|  | CpG1 | | | | |
| Deep muscle – convex side | 2.83 | 7.57 | 4.53 | 1.31 | 0.08^a^ |
| Deep muscle – concave side | 2.99 | 7.95 | 4.82 | 1.07 |  |
|  | CpG2 | | | | |
| Deep muscle – convex side | 2.97 | 6.46 | 5.05 | 0.94 | 0.08^b^ |
| Deep muscle – concave side | 2.89 | 7.99 | 5.37 | 1.24 |  |
|  | CpG3 | | | | |
| Deep muscle – convex side | 2.50 | 6.13 | 3.88 | 0.86 | **0.02^a^** |
| Deep muscle – concave side | 2.82 | 5.88 | 4.15 | 0.70 |  |
|  | CpG4 | | | | |
| Deep muscle – convex side | 2.61 | 4.13 | 3.46 | 0.58 | **0.001^a^** |
| Deep muscle – concave side | 1.64 | 5.93 | 4.06 | 1.07 |  |
|  | CpG5 | | | | |
| Deep muscle – convex side | 3.90 | 3.07 | 0.66 | 3.90 | **0.002^b^** |
| Deep muscle – concave side | 5.63 | 3.57 | 1.00 | 5.63 |  |
|  | CpG6 | | | | |
| Deep muscle – convex side | 3.12 | 2.63 | 0.41 | 3.12 | **0.007^a^** |
| Deep muscle – concave side | 4.59 | 2.97 | 0.67 | 4.59 |  |
|  | CpG7 | | | | |
| Deep muscle – convex side | 4.55 | 2.84 | 0.75 | 4.55 | **0.01^b^** |
| Deep muscle – concave side | 4.78 | 3.22 | 0.82 | 4.78 |  |
|  | CpG8 | | | | |
| Deep muscle – convex side | 3.17 | 2.40 | 0.39 | 3.17 | **0.03^b^** |
| Deep muscle – concave side | 4.44 | 2.63 | 0.62 | 4.44 |  |
|  | CpG9 | | | | |
| Deep muscle – convex side | 2.43 | 1.98 | 0.35 | 2.43 | **0.005^a^** |
| Deep muscle – concave side | 3.14 | 2.22 | 0.59 | 3.14 |  |
|  | CpG10 | | | | |
| Deep muscle – convex side | 4.84 | 2.88 | 0.90 | 4.84 | 0.16^b^ |
| Deep muscle – concave side | 4.79 | 3.12 | 0.82 | 4.79 |  |
|  | CpG11 | | | | |
| Deep muscle – convex side | 4.16 | 2.22 | 0.65 | 4.16 | 0.21^a^ |
| Deep muscle – concave side | 3.69 | 2.33 | 0.61 | 3.69 |  |
|  | CpG12 | | | | |
| Deep muscle – convex side | 9.51 | 5.71 | 1.52 | 9.51 | 0.20^b^ |
| Deep muscle – concave side | 8.66 | 6.03 | 1.20 | 8.66 |  |
|  | CpG13 | | | | |
| Deep muscle – convex side | 5.74 | 3.46 | 0.87 | 5.74 | 0.56^b^ |
| Deep muscle – concave side | 5.24 | 3.57 | 0.89 | 5.24 |  |
|  | CpG14 | | | | |
| Deep muscle – convex side | 6.83 | 4.51 | 0.86 | 6.83 | 0.27^a^ |
| Deep muscle – concave side | 7.10 | 4.69 | 0.92 | 7.10 |  |
|  | CpG15 | | | | |
| Deep muscle – convex side | 2,29 | 5,02 | 3,32 | 0,63 | 0.46^b^ |
| Deep muscle – concave side | 2,17 | 4,70 | 3,41 | 0,58 |  |
|  | CpG16 | | | | |
| Deep muscle – convex side | 4,83 | 9,32 | 6,54 | 1,21 | 0.13^a^ |
| Deep muscle – concave side | 4,90 | 8,96 | 6,82 | 0,89 |  |

Min – minimum; Max – maximum; ^a^ – Wilcoxon signed-rank test; ^b^ – dependent t-test

Table S2. Methylation level [%] of 16 CpGs within *ESR2* exon 0N (N=29).

|  | Min | Max | Mean | SD | *P*-value |
| --- | --- | --- | --- | --- | --- |
|  | CpG1 | | | | |
| Deep muscle – convex side | 2,96 | 6,63 | 4,87 | 1,28 | 0.89^a^ |
| Deep muscle – concave side | 2,66 | 7,06 | 4,84 | 1,15 |  |
|  | CpG2 | | | | |
| Deep muscle – convex side | 2,48 | 7,73 | 4,91 | 1,68 | 0.52^a^ |
| Deep muscle – concave side | 2,30 | 7,40 | 5,20 | 1,38 |  |
|  | CpG3 | | | | |
| Deep muscle – convex side | 2,31 | 4,41 | 3,62 | 0,78 | 0.24^a^ |
| Deep muscle – concave side | 1,71 | 6,10 | 3,84 | 1,17 |  |
|  | CpG4 | | | | |
| Deep muscle – convex side | 2,50 | 8,26 | 5,24 | 1,87 | 0,92^a^ |
| Deep muscle – concave side | 2,08 | 9,17 | 5,34 | 1,73 |  |
|  | CpG5 | | | | |
| Deep muscle – convex side | 2,68 | 11,92 | 6,69 | 2,71 | 0.73^b^ |
| Deep muscle – concave side | 2,87 | 9,81 | 6,51 | 2,09 |  |
|  | CpG6 | | | | |
| Deep muscle – convex side | 2,48 | 9,37 | 5,62 | 2,28 | 0.97^a^ |
| Deep muscle – concave side | 2,31 | 9,46 | 5,70 | 2,06 |  |
|  | CpG7 | | | | |
| Deep muscle – convex side | 1,37 | 5,04 | 2,95 | 1,24 | 0.72^a^ |
| Deep muscle – concave side | 1,35 | 5,84 | 3,08 | 1,10 |  |
|  | CpG8 | | | | |
| Deep muscle – convex side | 1,68 | 5,76 | 4,14 | 1,43 | 0.51^a^ |
| Deep muscle – concave side | 1,81 | 8,90 | 4,42 | 1,76 |  |
|  | CpG9 | | | | |
| Deep muscle – convex side | 1,80 | 5,99 | 3,43 | 1,29 | 0.73^a^ |
| Deep muscle – concave side | 1,57 | 5,12 | 3,33 | 1,07 |  |
|  | CpG10 | | | | |
| Deep muscle – convex side | 1,83 | 5,75 | 3,62 | 1,27 | 0.63^a^ |
| Deep muscle – concave side | 1,72 | 6,63 | 3,58 | 1,12 |  |
|  | CpG11 | | | | |
| Deep muscle – convex side | 2,21 | 9,63 | 4,91 | 2,24 | 0.69^a^ |
| Deep muscle – concave side | 2,09 | 8,36 | 4,71 | 1,55 |  |
|  | CpG12 | | | | |
| Deep muscle – convex side | 3,17 | 5,39 | 4,53 | 0,82 | 0.25^a^ |
| Deep muscle – concave side | 2,76 | 7,15 | 4,81 | 1,13 |  |
|  | CpG13 | | | | |
| Deep muscle – convex side | 2,66 | 9,74 | 5,25 | 2,08 | 0.71^a^ |
| Deep muscle – concave side | 2,72 | 6,78 | 5,06 | 1,30 |  |
|  | CpG14 | | | | |
| Deep muscle – convex side | 4,57 | 7,21 | 6,23 | 1,03 | 0.11^a^ |
| Deep muscle – concave side | 4,28 | 10,38 | 6,69 | 1,51 |  |
|  | CpG15 | | | | |
| Deep muscle – convex side | 3,42 | 4,62 | 4,20 | 0,53 | 0.12^a^ |
| Deep muscle – concave side | 3,12 | 4,93 | 4,29 | 0,68 |  |
|  | CpG16 | | | | |
| Deep muscle – convex side | 1,63 | 3,90 | 2,69 | 0,52 | 0.12^b^ |
| Deep muscle – concave side | 2,02 | 4,10 | 2,88 | 0,54 |  |
|  | CpG17 | | | | |
| Deep muscle – convex side | 2,19 | 8,66 | 4,76 | 1,76 | 0.67^a^ |
| Deep muscle – concave side | 2,74 | 6,09 | 4,64 | 1,06 |  |
|  | CpG18 | | | | |
| Deep muscle – convex side | 1,85 | 6,01 | 3,61 | 1,12 | 0.80^a^ |
| Deep muscle – concave side | 1,62 | 5,27 | 3,59 | 0,99 |  |
|  | CpG19 | | | | |
| Deep muscle – convex side | 3,54 | 11,37 | 6,47 | 2,23 | 0.93^a^ |
| Deep muscle – concave side | 4,12 | 8,65 | 6,44 | 1,49 |  |

Min – minimum; Max – maximum; ^a^ – Wilcoxon signed-rank test; ^b^ – dependent t-test
